# Assessment of *pfcrt* 72-76 haplotypes eight years after chloroquine withdrawal in Kinshasa, Democratic Republic of Congo

**DOI:** 10.1186/1475-2875-12-459

**Published:** 2013-12-20

**Authors:** Dieudonné Makaba Mvumbi, Raphael Boreux, Rosalie Sacheli, Mvumbi Lelo, Bobanga Lengu, Situakibanza Nani-Tuma, Pierrette Melin, Kayembe Ntumba, Kalala Lunganza, Patrick DeMol, Marie-Pierre Hayette

**Affiliations:** 1 Biochemistry and Molecular Biology Unit, Department of Basic Sciences, School of Medicine, University of Kinshasa, BP 190 KIN XI, Kinshasa, DR Congo; 2 Department of Clinical Microbiology, University Hospital of Liège, Liège, Belgium; 3 Department of Tropical Medicine and Infectious Disease, School of Medicine, University of Kinshasa, Kinshasa, DR Congo; 4 Department of Internal Medicine, School of Medicine, University of Kinshasa, Kinshasa, DR Congo

**Keywords:** Malaria, *pfcrt*, Drug resistance, RT-PCR, Sequencing, DR Congo

## Abstract

**Background:**

In 2001, the World Health Organization (WHO) has recommended the use of artemisinin-based combination therapy (ACT) as the first-line treatment of uncomplicated malaria cases, as monotherapies had become ineffective in many parts of the world. As a result, the Democratic Republic of Congo (DRC) withdrew chloroquine (CQ) from its malaria treatment policy in 2002 and an artesunate (AS)-amodiaquine (AQ) combination became the ACT of choice in DRC in 2005. AQ-resistance (AQR) has been reported in several parts of the world and mutations in codons 72-76 of the *Plasmodium falciparum* chloroquine-resistance transporter (*pfcrt*) gene have been strongly correlated with resistance, especially mutations encoding the SVMNT haplotype. This haplotype was first identified in Southeast Asia and South America but was recently reported in two African countries neighbouring DRC. These facts raised two questions: the first about the evolution of CQ resistance (CQR) in DRC and the second about the presence of the SVMNT haplotype, which would compromise the use of AQ as a partner drug for ACT.

**Methods:**

A total of 213 thick blood films were randomly collected in 2010 from a paediatric clinic in Kinshasa, DRC. Microscopy controls and real-time polymerase chain reaction (RT-PCR) were performed for *Plasmodium* species identification. Haplotypes of the *pfcrt* gene were determined by sequencing.

**Results:**

The K76T mutation was detected in 145 out of 198 *P. falciparum*-positive samples (73.2%). In these 145 resistant strains, only the CVIET haplotype was detected.

**Conclusions:**

This study is the first to assess the molecular markers of resistance to CQ and AQ after the introduction of ACT in DRC. The results suggest first that CQR is decreasing, as wild-type *pfcrt* haplotypes were found in only 26.8% of the samples and secondly that the SVMNT haplotype is not yet present in Kinshasa, suggesting that AQ remains valid as a partner drug for ACT in this region.

## Background

In 2001, the World Health Organization (WHO) has recommended the use of artemisinin-based combination therapy (ACT) as the first-line treatment of uncomplicated malaria cases because monotherapies had become ineffective in many parts of the world due to the spread of *Plasmodium falciparum* strains resistant to almost all anti-malarial drugs in use [1].

Resistance to chloroquine (CQ), the drug that had been widely used for decades for malaria treatment [2], has been well characterized and it is now established that a mutation (K76T) occurring in the *P. falciparum* chloroquine-resistance transporter (*pfcrt*) gene is the key element [3-5]. Nevertheless many other mutations have been described in this gene [6].

Childs and colleagues were the first to show *in vitro* cross-resistance between amodiaquine (AQ) or its active metabolite and CQ [7]. Because of their pharmacological similarity, CQ and AQ were supposed to have the same molecular mechanism of resistance. Furthermore, Ochong et al., by analysing samples from a clinical trial held in southern Sudan, demonstrated a link between the K76T mutation and *in vivo* AQ resistance [8]. A few years later, other studies performed on the *pfcrt* gene revealed that some haplotypes defined by the aminoacids sequence at positions 72-76 of the CRT protein were related to a particular resistance profile and also to a specific geographical distribution. Sa et al. analysed reference strains and provided the first *in vitro* evidence that one of these haplotypes, the SVMNT (Ser-Val-Met-Asn-Thr), was strongly correlated with resistance to desethylamodiaquine (DEAQ), the active metabolite of AQ [9]. One year later, *in vivo* evidence of this link has been given by Beshir et al. who found that the presence of the SVMNT haplotype was sufficient to confer AQ resistance [10].

This haplotype was initially discovered in Asia and in South America [11,12], but it has recently been found in two African countries: first in Tanzania in 2006 [13], then in 2010 in Angola [14]. Both countries border the Democratic Republic of Congo (DRC), where CQ, after being widely used, was officially abandoned in 2002 to be replaced first by sulphadoxine-pyrimethamine (SP) and then by an artesunate-amodiaquine (AS-AQ) combination in 2005 [15].

This raised questions about the presence of the SVMNT haplotype in the DRC, which would jeopardize the use of AQ as a partner drug for ACT. These questions were addressed in this study.

## Methods

### Sample collection

A total of 213 thin and thick blood films (TBF) were randomly selected in April 2010 from the laboratory archives of the St Marc Paediatric Clinic of Masina (Kinshasa). All the smears were positive using microscopic recordings as selection criteria. The blood samples had been collected between January and March 2010 from children under five years who were suspected of malaria.

### Malaria diagnosis

All blood smears underwent microscopic control performed by two experienced microscopists and the results were confirmed by real-time polymerase chain reaction (RT-PCR).

### DNA extraction

The whole TBF was used for DNA extraction, leaving the thin smear for microscopic reading. Each TBF was first immersed in xylene for 10 s to avoid contamination between samples and to dissolve any residual oil from previous microscopic examinations. Then it was scratched out with a sterile scalpel as described previously by Cnops et al. [16]. The scraped material was transferred into a 1.5 ml sterile Eppendorf tube containing 100 μl of PBS and then used as a template for a semi-automated extraction using the Maxwell® 16 Cell LEV DNA purification Kit (Promega, USA). A DNA internal extraction and inhibition control was used to follow the extraction procedure (DIAcontrolDNA®, Diagenode, Belgium). DNA was stored at -20°C before processing.

### Real-time PCR

The RT-PCR method for *Plasmodium* sp*.* identification was used as previously described [17]. Briefly, a four-primer PCR was used in two duplex reactions including a couple of forward primers (*Pfal* + *Pviv* and *Pmal* + *Pova*) with a universal reverse primer (plasmo2). The probes and primers were designed to detect genes of the small subunit 18S rRNA of *Plasmodium* species (Table 1). The mix consisted of: 12.5 μl of 2X Taqman Universal PCR Master Mix (Applied Biosystems, USA), 200 nM of all primers and probes except *P. vivax* at 100 nM (primers and probe), 2.5 μl of Internal Control primers and probes and the rest of water to make a total volume of 25 μl including 5 μl of DNA. PCR tests were run on a 7500 Fast Instrument (Applied Biosystems) and in the presence of positive controls (provided by the Parasitology Unit, Institute of Tropical Medicine, Antwerp and the Laboratory of Clinical Microbiology, University Hospital of Liège). PCR conditions were as follows: 2 min at 95°C, followed by 50 cycles of 15 s at 95°C and 60 s at 60°C.

**Table 1:** Primer and probe sequences

**RT-PCR**	**Forward primer (plasmo1)**	**Reverse primer (plasmo2)**	**Probes**
***Pfal***	5′-CTAGGTGTTGGATG-3′	5′-TTATGAGAAATCAAAGTCTTTGGGTT-3′	5′-FAM-AGCAATCTAAAAGTCACCTCGAAAGATGACT-DQ-3′
***Pova***	5′-CGACTAGGTTTTGGATG-3′		5′-VIC-CGAAAGGAATTTTCTTATT-DQ-3′
***Pviv***	5′-GACTAGGCTTTGGATG-3′		5′-VIC-AGCAATCTAAGAATAAACTCCGAAGAGAAAATTCT-DQ-3′
***Pmal***	5′-GACTAGGTGTTGGATG-3′		5′-FAM-CTATCTAAAAGAAACACTCAT-DQ-3′
**PCR/genotyping**	**Forward primer**	**Reverse primer**	
***Pfcrt***	5′-GTTCTTGTCTTGGTAAATGTGCTCA-3′	5′-CAATT TTGTTTAAAGTTCTTTTAGCAA-3′	

Legend: Pfal: *P. falciparum*; Pova: *P.ovale*; Pviv: *P. vivax*; Pmal: *P. malariae;* DQ: Darkquencher.

### *Pfcrt* genotyping

The procedure previously described by Ménard et al. [18] was modified by adding an M13 fragment on the 5′ ends of the *pfcrt* primers (Table 1) to amplify a 154 bp fragment that included the *pfcrt* codons in positions 72-76 of all of the *P. falciparum*-positive samples. PCR tests were run on a Hybaid PX2 (ThermoFischer Scientific, USA) under the following conditions: 95°C for 10 min, followed by 45 cycles of 20 s at 95°C and 45 s at 60°C. Amplification was controlled on a microchip electrophoresis system using the MCE®-202 MultiNA (Shimadzu). Amplicons were purified on Sciclone G3 Automated Liquid Handling Workstation (Perkin Elmer, USA) using AgencourtCleanSEQ® kit (Agencourt Bioscience, USA).

Sequencing was performed on a 3130xl DNA sequencer (Applied Biosystems, USA).

Sequences were aligned using the GeneStudioTM® Professional software and were compared with the reference sequence of the CRT protein [GenBank: CAD50842.1] using the online BLASTx tool.

### Ethical considerations

This study has received the ethical approbation of the Ministry of Public Health of the DRC and of the Institutional Committee of the Faculty of Medicine, University of Kinshasa.

## Results

### Malaria diagnosis confirmation

RT-PCR lead to the detection of 202/213 (93.4%) positive samples for *P. falciparum* including three mixed infections (*P. falciparum* + *Plasmodium malariae*).

### *Pfcrt* haplotypes

*Pfcrt* genotyping was successfully performed for 198 *P. falciparum* samples from the 202 positive samples detected by RT-PCR. Among them, 145 K76T mutants (73.2%) were detected and they were all harbouring the CVIET haplotype. The wild strains had the CVMNK haplotype. No other haplotypes were detected.

## Discussion

Nearly ten years after the withdrawal of CQ in the first-line treatment of malaria in the DRC, CQR strains remain above the warning threshold established by WHO. However the prevalence of pfcrt 76 T mutants continues to decline as shown in previous studies performed in DR Congo where a slow reversion in sensitivity has been recorded. Indeed, the percentage of Pf resistant strains went from 100% in 2000 [19], to 93% in 2002 [20], 83.8% in 2008 [21] and 73.2% in the present study. Finally, over the last ten years, the prevalence of resistant strains has decreased by 26.8%. In other countries however, the decrease was much more significant like in Malawi where CQR fell from 85% to 13% in 8 years [22] after replacing CQ by SP or in Gabon with a drop of 55% (100% to 45%) in 6 years [23]. This fact could be explained by the large area covered by DRC that is more difficult to control, and by the relatively high cost of ACT compared to former monotherapies, resulting in a slower anti-malarial policy shift. Until 2007, CQ was still the most widely used anti-malarial drug in the DRC [24] although it was officially removed since 2002 and firstly replaced by SP. Aside from the efficiency with which new drug policies were implemented, malaria transmission intensity in the region and the use of many other anti-malarial drugs in addition to the national policy have been suggested to explain the difference in trend [25].

The CVIET haplotype was the only resistant haplotype identified in the present study. Indeed, it is the most prevalent haplotype among the five discovered to date in Africa and it seems that it has migrated from Southeast Asia to Africa via India [26,27]. Some studies have described this haplotype as a necessary but insufficient condition to confer AQ resistance [28,29]. Beshir et al. suggested that if the CVIET haplotype is highly prevalent, AQ can still be effective and further mutations on the *P. falciparum* multidrug resistance gene (*pfmdr1*) are required for the development of clinically significant AQ resistance [10].

The only existing results on 72-76 *pfcrt* haplotypes that are available for the DRC refer to samples collected in 2000. The study identified 100% of resistant strains and these were distributed as follows: 14/27 (51.8%) SVIET, 12/27 (44.4%) CVIET and 1/27 (3.7%) of CVMNT [18]. Studies on a larger scale should be more representative of the high prevalence of the SVIET haplotype, which appears to be rare elsewhere in the world. In fact, it was found before only once, in a study performed in Papua New Guinea [30].

Our results showed that the SVMNT haplotype is not yet present in Kinshasa suggesting that AQ remains valid as a partner drug for ACT. However, continuous monitoring is necessary because, initially absent from Africa, the SVMNT haplotype suddenly appeared in Tanzania in 2004, where it was not present the year before [13]. This fact shows how the emergence of a new haplotype can be spontaneous. This haplotype has been also found recently in Angola by Gama et al. who supposed that this haplotype may have been imported from Brazil [14]. Frosch et al. showed that Angola and Tanzania were among African countries where AQ was more extensively used than in other countries during the last decade [31]. This fact could have contributed to the selection of SVMNT resistant strains, as suggested by Sa and by others, who stipulated that AQ had a prominent role in the selection of parasites carrying the SVMNT haplotype [10,13,32,33]. In fact, SVMNT haplotype is predominant in countries where AQ was early and widely used [32].

Malaria is a disease of great geographical diversity and, as DRC is a very large country, the results of this study concern only the region of Kinshasa and further studies are needed.

## Conclusion

This study confirms the slow reverse in sensitivity to CQ in Kinshasa. This is the first to assess resistance to AQ through *pfcrt* haplotypes after CQ withdrawal, especially based on TBFs. AQ remains effective as a partner molecule for ACT in Kinshasa but monitoring of *P. falciparum* genomic resistance is mandatory.

## Abbreviations

ACT: Artemisinin-based combination therapy; AQ: Amodiaquine; AS: Artesunate; CQ: Chloroquine; DNA: Deoxyribonucleic acid; DRC: Democratic Republic of Congo; RT-PCR: Real-time polymerase chain reaction; SP: Sulphadoxine-pyrimethamine; TBF: Thick blood film; WHO: World health organization.

## Competing interests

The authors declare that they have no competing interest

## Authors’ contributions

MMD collected samples, carried out the DNA extraction, performed RT-PCR analysis and drafted the manuscript. RB and RS supervised genotyping analysis.ML, KL and SN conceived and designed the study protocol. BL participated in collecting samples. PM provided materials. MPH, KN and PDM supervised the study. All authors read and approved the manuscript.
